# Adiponectin: An Indicator for Metabolic Syndrome

**Published:** 2019-06

**Authors:** Somaye YOSAEE, Mahmoud KHODADOST, Alireza ESTEGHAMATI, John R. SPEAKMAN, Kurosh DJAFARIAN, Vida BITARAFAN, Farzad SHIDFAR

**Affiliations:** 1. Department of Nutrition, School of Health, Larestan University of Medical Sciences, Larestan, Iran; 2. Department of Nutrition, Emam Reza Teaching Hospital, Larestan University of Medical Sciences, Larestan, Iran; 3. Department of Epidemiology, School of Public Health, Shahid Beheshti University of Medical Sciences, Tehran, Iran; 4. Gerash University of Medical Sciences, Gerash, Iran; 5. Endocrinology and Metabolism Research Center (EMRC), Vali-Asr Hospital, Tehran University of Medical Sciences, Tehran, Iran; 6. State Key Laboratory of Molecular Developmental Biology, Institute of Genetics and Developmental Biology, Chinese Academy of Sciences, Beijing, China; 7. Institute of Biological and Environmental Sciences, University of Aberdeen, Scotland, UK; 8. Department of Clinical Nutrition, School of Nutritional Sciences and Dietetics, Tehran University of Medical Sciences, Tehran, Iran; 9. Adelaide Medical School and National Health and Medical Research Council of Australia (NHMRC), Center of Research Excellence in Translating Nutritional Science to Good Health, University of Adelaide, Adelaide, Australia; 10. Department of Nutritional Sciences, School of Public Health, Iran University of Medical Sciences, Tehran, Iran

**Keywords:** Leptin, Adiponectin, Metabolic syndrome, Fat mass

## Abstract

**Background:**

Metabolic syndrome (MetS), a cluster of cardiometabolic risk factors, consider as a manifestation of obesity. However, a proportion of obese patients do not develop MetS. The aim of our study was to determine whether concentration of plasma adiponectin and leptin differ between metabolic unhealthy obese (MUO) patients and comparable age- and sex-matched control groups.

**Methods:**

In this case-control study, we assigned 51 obese patients with MetS (MUO) in cases group and 102 metabolic healthy obese (MHO) and normal weight metabolic healthy subjects matched for age and gender to cases in control groups. The study was conducted between December 2014 and February 2016 in the Endocrinology Research Center of Tehran University of Medical Sciences, Tehran, Iran. We measured serum adiponectin, leptin, their ratio, and body composition in all subjects.

**Results:**

No significant differences were observed between MHO and MUO in term of total fat mass and trunk fat (*P*>0.05). Compared to MHO and normal weight metabolic healthy subjects, MUO subjects had lower levels of plasma adiponectin (*P*<0.001) and lower plasma adiponectin to leptin ratio (*P*<0.001) and a higher level of plasma leptin (*P*<0.002). A Receiver Operator Characteristic curve was used to identify the ability of adiponectin and leptin level to predict the MetS. The area under the Receiver Operator Characteristic curve was 0.66 (*P*<0.01), 0.73 (*P*<0.001) and 0.75 (*P*<0.001) for leptin, adiponectin, and adiponectin/leptin ratio levels respectively.

**Conclusion:**

Our study introduced adiponectin and leptin as indicator of MetS and obesity respectively.

## Introduction

Metabolic syndrome (MetS) or insulin resistance syndrome is defined as a cluster of cardiometabolic risk factors including of the following characteristics: abdominal adiposity, dyslipidemia, high blood pressure, fasting glucose, or insulin resistance (1). MetS is one of the leading global public health issues (2). The number of patients with MetS in developed and developing countries is expanding (3, 4). Its prevalence varies between 15% and 40% (5), with a higher rate in developing countries (5). MetS poses an increased risk of coronary heart disease (CHD), type 2 diabetes mellitus (DM2), and other cardiometabolic diseases (6).

In spite of various pathogenic mechanisms proposed, the etiology and concept of the MetS still remains obscure and controversial and is under intense investigation (7). Although the prevalence of MetS increases with obesity, MetS can be present in lean individuals; furthermore, a proportion of obese patients do not affected by MetS so called “healthy obese phenomenon” (8). Recent research introduced adipocytes-derived hormones, as mediators linking adipocytes-dysfunction to MetS (9, 10). These so-called adipokines are important determinants of insulin resistance and obesity (11). Adiponectin and leptin are two most widely studied adipocytokines in relation to MetS (9, 10).

Adiponectin, the most abundant anti-atherogenic and anti-inflammatory adipocytokine found in circulation has direct effects on glucose and lipid metabolism, improves insulin sensitivity and central fat distribution (12, 13). Adiponectin levels are inversely correlated with visceral adiposity (14). A lower level of adiponectin is associated with insulin resistance, obesity, MetS and CVD (15, 16). Low level of circulating adiponectin may be used as a possible biomarker for MetS (17). On the other hand, Leptin as an anti-obesity adipocytokine plays critical roles in regulating food intake, maintaining energy expenditure and body weight (15). Serum leptin levels in patients with MetS are higher than those in healthy controls (18). Increased leptin level predicts metabolic syndrome development independent of obesity (19). In fact, adiponectin and leptin levels show an inverse correlation with each other (19). Therefore, to be integrated their critical roles in MetS, adiponectin/leptin ratio has been identified to be useful serum markers for diagnosis of MetS (20, 21), and even to predict of CV outcome (22). The aim of this study was to determine adiponectin and leptin profiles and their ratio from serum in MUO patients compared to age and gender-matched control groups.

## Materials and Methods

### Subjects

The study was a case-control study comprising 51 metabolically unhealthy obese (MUO) as cases and 102 metabolically healthy obese (MHO) and normal weight metabolic healthy as control groups (51 subjects in each group). The study was conducted between December 2014 and February 2016 in Tehran the Endocrinology Center of Tehran University of Medical Sciences, Tehran, Iran. Fifty-one patients newly diagnosed with MetS and never receiving medications were included in this study as MUO group. The two control groups included 51 MHO and 51 normal weight metabolic healthy subjects (healthy). Subjects were matched for age and gender. The sample size was calculated based on previous study with the minimum differences in waist to hip ratio by 0.05 and standard deviation by 0.14, with the power of 80% and α error of 5%, we determine the sample size of 50 in every case and control groups.

Informed consent was obtained from all the subjects participating in the study. The research protocol of the study was approved by Scientific Research Committee of Iran University of medical science (93-02-27-24976).

### Diagnostic criteria for the metabolic syndrome

Diagnostic criteria recommended National Cholesterol Education Program (NCEP) - Adult Treatment Panel (ATP III) (NCEP ATPIII) was used to define MetS.

Waist circumference (WC) was measured between the lower rib margin and the iliac crest by a flexible tape measure after normal expiration. Body weight was measured while participants wore light clothing using calibrate scale to an accuracy of ±0.5 kg (23, 24). Standing height was measured with a standard stadiometer to the nearest 0.1 cm, measuring the participants without shoes, erect and with the back against a flat surface (24). Systolic and diastolic blood pressure was measured on the non-dominant brachial artery in the sitting position after the subject resting for at least 10 min by a specialist nurse (23, 24). Blood pressure was measured twice separately over a 3-min interval. The average blood pressure considered as blood pressure value. Levels of TG, HDL-c, and FBS were evaluated from patients medical chart records.

### Exclusion and inclusion criteria

Having a history of coronary artery disease, acute or chronic renal failure, acute infection within the previous seven days, acute or chronic hepatic failure, hematological disorder, presence of any chronic inflammatory and autoimmune disease, and any known malignancy, hormone therapy, taking phytoestrogens or drug intended to decrease lipid level. Other non-pathological exclusion criteria included pregnancy, breastfeeding, post-menopause, smoking, professional athlete, uncontrolled thyroid disorder, use of medications for dyslipidemia or hypertension, hypnotics, sedatives and immunosuppressive or having a special diet for any reason prescribed by the clinic dietitian.

The inclusion criteria included age range 20–55 yr and provided written consent of participation.

### Assay for leptin and adiponectin

Peripheral venous blood sample was drawn after more than 8 h of fasting overnight. Serum adipocytokine levels (leptin and adiponectin) were measurements by enzyme-linked immune-sorbent assay (ELISA) using a Mediagnost Kit (Reutlingen/Germany). The assay was conducted according to the manufactures instructions. Plasma samples required 310-fole dilution for adiponectin.

### Body composition measurement

Body composition component including were measured using 8-contact electrode method of Bio-Electrical Impedance Analysis (BIA) (model TANITA BC-418). For this purpose, the subjects were asked to empty their bladder prior to the testing and stand on metal footpads in bare feet and grasp a pair of electrodes fixed on a handle. There is a significant correlation regarding the data obtained from BIA and DXA (25).

### Statistical analysis

SPSS 16.0 software was used for basic statistical analysis (SPSS Inc., Chicago, IL, USA). Comparisons between three groups were carried out using the one-way ANOVA. When the result of the ANOVA test was significant, a LSD test was used to locate which of the means differed. Moreover, the analysis of covariance (ANCOVA) was used for adjustment on fat components in assessing the association between adiponectin and leptin level among the study groups. Receiver Operator Characteristic curve (ROC curves) was used for determining the appropriate cut off point and its sensitivity and specificity of adiponectin, leptin levels and also adiponectin/leptin ratio for predicting metabolic syndrome. Multiple linear regression analysis was used to assess the association between adiponectin and other study parameters. In addition, it was applied to identify whether leptin, adiponectin and its ratio were independently associated with MetS. A value of *P*<0.05 was accepted as statistically significant.

## Results

The characteristics and MetS components of our subjects with respect to study groups are summarized in Table 1.

**Table 1:** General characteristics, MetS parameters AND serum adiponectin, leptin and adiponectin/leptin ratio of subjects based on study groups

***Variable***	***MUO(51)***	***MHO(51)***	***Normal weight metabolic healthy (n=51)***	***Total (153)***	P-value^*^
	mean±SD	mean±SD	mean±SD	mean±SD	
Age (year)	37.47±6.5	36.94±6.4	35.93±6.3	36.78±6.4	0.47
Weight (Kg)	92.5±13.3^a^	89.1±13.7^a^	69.1±6.8^b^	83.6±15.58	<0.0001
Height (Cm)	171.49±7.7	172.2±7.03	171.1±6.8	171.6±7.1	0.74
WC (Cm)	105.09±8.1^a^	100.2±11.3^b^	87.02±7^c^	97.4±11.79	<0.0001
BMI (Kg/m^2^)	31.43±3.7^a^	29.9±3.6^b^	23.6±1.4^c^	28.3±4.5	<0.0001
SBP (mm-Hg)	121.2±11.7^a^	113.8±8.6^b^	111.3±12.2^b^	115.4±11.6	<0.0001
DBP (mm-Hg)	80.8±9.1^a^	75.7±6.6^b^	74.1±8.3^b^	76.8±8.5	<0.0001
FBS (mg/dl)	116.2±37.3^a^	97.8±20.4^b^	95.2±7.6^b^	103.1±26.4	<0.0001
TG (mg/l)	266.3±203.6^a^	131.05±87.4^b^	117.5±67.8^b^	171.6±149.02	<0.0001
HDL-c (mg/dl)	50.3±8.3^a^	54.3±5.6^b^	55.09±9.2^b^	53.2±8.09	0.006
MetS parameters	3.31±0.61^a^	1.05±0.75^b^	0.8±0.63^b^	1.7±1.3	<0.0001
Leptin(ng/ml)	14.06±12.4^a^	11.2±9.3^a^	7.09±7.1^b^	10.8±10.2	0.002
Adiponectin(μg/ml)	4.85±1.8^a^	6.7±2.8^b^	7.25±3.2^b^	6.25±2.8	<0.0001
Adiponectin/leptin	0.58±0.56^a^	1.17±1.3^b^	2.01±2.1^c^	1.24±1.6	<0.0001

Values are analyzed by one–way ANOVA, values are mean ± SD.

Dissimilar values (a, b, c) of each row are significantly different

In one way analysis of variance (one way-ANOVA), leptin was significantly different between obese subjects with/without Mets groups (MHO and MUO groups) and normal weight metabolic healthy subjects. MUO patients had higher levels of leptin (14.06±12.4) compared to those without MetS (11.2±9.3 Vs 7.09±7.1 in MHO and normal weight metabolic healthy respectively; *P*=0.002) (Table 1). Moreover, in analysis of covariance (ANCOVA), after adjustment for fat content, there is still a significant association between leptin and study groups (*P*=0.015), so that the mean of leptin in MUO patients was significantly lower compared to average concentrations in the normal weight metabolic healthy group (Tables 2, 3).

**Table 2:** Analysis of covariance for assessing association between adiponectin and leptin with study groups

***Variable***	***Source***	***Sum of squares***	***df***	***Mean square***	***F***	***P-value***
Adiponectin (μg/ml)	Intercept	212.63	1	212.63	31.00	<0.001
	Fat mass	24.50	1	24.50	3.57	0.061
	Study groups	184.39	2	92.19	13.44	<0.001
	Error	1008.20	147	6.85		
	Total	7094.24	151			
Leptin (ng/ml)	Intercept	3382.31	1	3382.31	90.43	<0.001
	Fat mass	8949.26	1	8949.26	239.28	<0.001
	Study groups	325.48	2	162.74	4.35	0.015
	Error	5497.85	147	37.40		
	Total	33446.96	151			
Adiponectin/leptin	Intercept	128.63	1	128.63	71.72	<0.001
	Fat mass	59.41	1	59.41	33.12	<0.001
	Study groups	9.85	2	4.92	2.74	0.067
	Error	263.63	147	1.79		
	Total	608.73	151			

**Table 3:** Estimated marginal means of adiponectin and leptin among case and control groups

***Variable***	***MUO(51)***	***MHO(51)***	***Normal weight metabolic healthy (n=51)***	P-value^*^
	mean±SD	mean±SD	mean±SD	
Leptin(ng/ml)	10.51±6.28^a^	9.07±6.14^a^	13.01±6.78^b^	0.015
Adiponectin(μg/ml)	4.66±2.71^a^	6.57±2.64^b^	7.56±2.85^b^	<0.001
Adiponectin/leptin	0.87±1.35^a^	1.34±1.35 ^a^	1.53±1.42^b^	0.067

Values are analyzed by analysis of covariance (ANCOVA), values are mean ± SD.

Dissimilar values (a, b, c) of each row are significantly different

The serum adiponectin level was significantly different between MUO and non-MetS groups (MHO and normal weight metabolic healthy groups). MUO patients (4.85±1.8) had significantly (*P*<0.0001) lower adiponectin level compared to MHO group (6.7±2.8) and normal weight metabolic healthy group (7.25±3.2) (Table 1). In analysis of covariance (ANCOVA), after adjustment for fat content, there was still a significant association between adiponectin and study groups (*P*<0.001), so that the mean of adiponectin in MUO patients was significantly lower compared to average concentrations in the MHO and normal weight metabolic healthy groups (Tables 2, 3). Adiponectin/leptin ratio was 0.58±0.56 in the MUO group and 1.17±1.3 in MHO and 2.01±2.1 in normal weight metabolic healthy groups (*P*<0.0001) (Table 2). However, in analysis of covariance (ANCOVA) after adjustment for fat content, the mean of Adiponectin/leptin ratio in MUO patients was lower than the average concentrations in the MHO and normal weight metabolic healthy groups, but this association was not statistically significant (*P*=0.067) (Tables 2, 3).

A Receiver Operator Characteristic curve was used to identify the ability of leptin, adiponectin and their ratio levels to predict the MetS (Table 4).

**Table 4:** Area under the ROC curve, sensitivity, specificity and cutoff value for leptin, adiponectin and adiponectin/leptin as predictor MetS

***Variable***	***AUC (95% CI)***	***Sensitivity***	***Specificity***	***P-value***
Leptin (8.1)	0.66 (0.57–0.75)	67.0%	64.0%	<0.001
Adiponectin (5.75)	0.73 (0.65–0.81)	73.0%	61.0%	<0.0001
Adiponectin/leptin (0.726)	0.75 (0.67–0.83)	75.0%	61.0%	<0.0001

AUC: area under the curve; CI: confidence interval

**Fig. 1:**
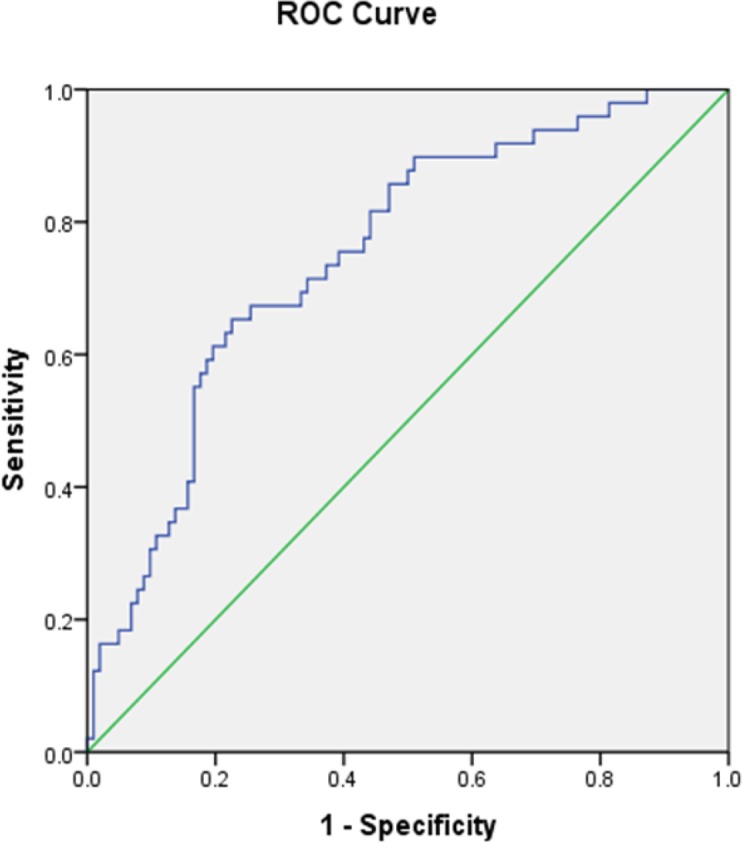
ROC curves for adiponectin/leptin ratio, as predictors of MetS Criterion<0.726 AUC=0.75 95% CI=0.67–0.83 SE=0.04 *P*= 0.0001

ROC curves and area under the curve (AUC) for the ability of adiponectin/leptin ratio to predict MetS are shown in Fig. 1. The cutoff for leptin, adiponectin and their ratio were 8.1 (ng/ml), 5.75 (μg/ml) and 0.726, respectively. The area under the Receiver Operator Characteristic curve was 0.66, 0.73 and 0.75 for leptin, adiponectin and their ratio levels, respectively (*P*>0.05).

Linear regression analysis was used to assess association between adiponectin and other study parameters (Table 5). In linear regression analysis with crude model (model 1), there was a significant association between adiponectin and gender (*P*=0.001), study groups (*P*<0.001), weight (*P*<0.001), waist circumference (*P*<0.001), BMI (*P*=0.001), SBP (*P*=0.004) FBS (*P*=0.001), TG (*P*=0.002), HDL (*P*=0.006) and number of MetS component (*P*<0.001).

**Table 5:** Multiple linear regression for assessing association between adiponectin and related factors

***Variables***	***Model 1^a^***		***Model 2^b^***		***Model 3^c^***	
	***β***	***P-value***	***β***	***P-value***	***β***	***P-value***
Leptin(ng/ml)	0.027	0.228	−0.043	0.159		
Age(yr)	−0.052	0.146				
Gender	2.2	0.001			2.073	0.001
groups^*^	−1.20	<0.001	−1.15	<0.001	−1.025	<0.001
Weight (cm)	−0.056	<0.001	−0.044	0.004		
Waist circumference (cm)	−0.07	<0.001	−0.053	0.008		
BMI (Kg/m^2^)	−0.169	0.001	−0.158	0.001		
SBP (mm-Hg)	−0.057	0.004	−0.42	0.29	−0.017	0.042
FBS (mg/dl)	−0.029	0.001	−0.27	0.001		
TG (mg/l)	−0.005	0.002	−0.004	0.011		
HDL-c (mg/dl)	0.077	0.006	0.057	0.042		
Number of MetS component	−0.678	<0.001	−0.637	<0.001		
Fat mass (%)	−0.003	0.922	−0.106	0.01		
Fat free mass (%)	0.003	0.922	0.106	0.01		
Trunk fat (%)	−0.035	0.293	−0.084	0.019		

a Crude model

b Adjusted for demographic variables such as sex, age, marital status and educational level.

c Multivariate backward linear regression model.

*: study groups including; 1: normal weight metabolic healthy, 2: MHO, 3: MUO

Moreover, in model 2, with adjusting for demographic variables, there was a significant association between adiponectin and study groups (*P*<0.001), weight *(P*=0.004), waist circumference (*P*=0.008), BMI (*P*=0.001), FBS (*P*=0.001), TG (*P*=0.011), HDL (*P*=0.0042), number of MetS component (*P*<0.001), fat mass (*P*=0.01), fat free mass (*P*=0.01) and trunk fat (*P*=0.019). In multiple linear regression analysis with backward method (model 3), there was a significant association between adiponectin and gender (*P*=0.001), study groups (*P*<0.001) and SBP (*P*<0.042). With increasing one unit in SBP, the adiponectin was decrease by 0.017.

**Fig. 2:**
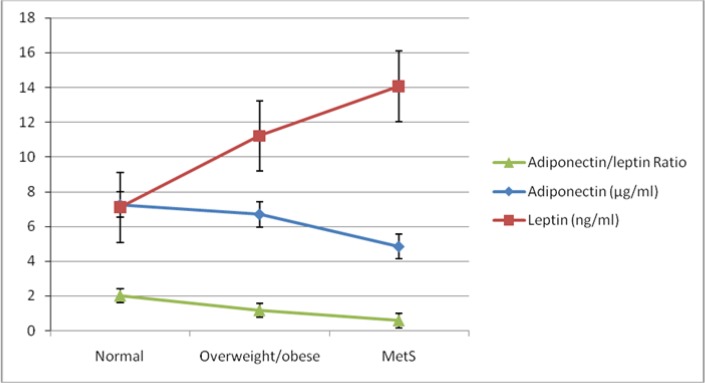
Trend of adiponectin, leptin and adiponectin/leptin ratio according to case and control groups

In addition, with increasing one unit in study groups (normal weight metabolic healthy groups in compare with MHO and MUO groups); the adiponectin was decrease by 1.025. The MetS group of study significantly has a low level of adiponectin compared to other groups of study so that there is a decreasing trend of adiponectin level in study groups (Fig. 2).

**Fig. 3:**
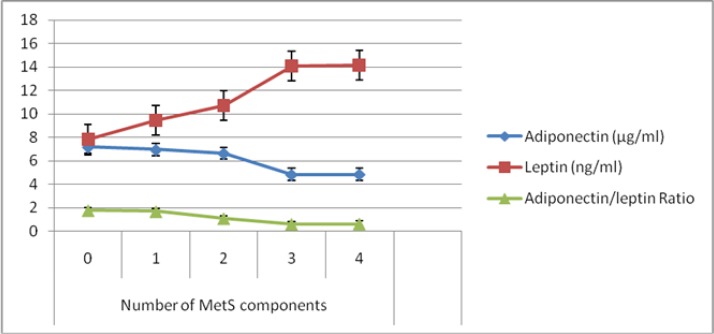
Trend of adiponectin, leptin and adiponectin/leptin ratio according to MetS components

The results of linear regression showed that there is significant association between adiponectin/leptin ratio with number of MetS components (*P*<0.001) and study groups (*P*<0.001). With increasing one unit in number of MetS components, the adiponectin/leptin ratio was decreased by 0.366, so that there is a decreasing trend of adiponectin/leptin ratio according to number of MetS components and study groups (Figs. 2 and 3). Moreover, there is significant association between leptin and number of MetS components (*P*=0.003) and study groups (*P*=0.001).

With increasing one unit in number of MetS components, the leptin were increase by 1.87, so that there is an increasing trend of leptin according to number of MetS components and study groups (Figs. 2 and 3).

## Discussion

In the present study, we investigated the different levels of circulating adiponectin and leptin and adiponectin/leptin ratio among MHO and MUO patients as well as normal weight metabolic healthy subjects in Iranian adults. There were significant differences in the plasma adiponectin level between obese subjects with and without MetS (MHO and MUO groups). Serum adiponectin was significantly lower in the MUO subjects than MHO as well as normal weight metabolic healthy subjects. This confirms the results of other studies (26, 27). In our study, the serum adiponectin level explained more specifically “healthy obese phenomenon” than leptin. Our data showed that the low levels of adiponectin are associated with a considerable rise in FBS, TG, BP, WC, BMI, number of MetS components, MetS development and declined in HDL-c. These findings remain statistically significant after adjusting for leptin and body composition, indicating that adiponectin can partly explain the healthy obese phenomenon and protect against MetS development in MHO subjects. Higher adiponectin levels were correlated with a favorable metabolic status and inflammatory profile (28).

In the present study, serum, leptin in MUO patients was similar to that in the MHO group. Supporting this hypothesis originates from the studies that report no significant difference in the serum leptin levels between MHO and MUH subjects (28, 29). Furthermore, the obtained data is consistent with the results of other studies that also showed that an elevated leptin level potentiated insulin resistance and hypertension while adiponectin, on the contrary, possessed cardio-protective effects (30). In addition present finding is in agreement with some reports that demonstrated lower leptin/adiponectin ratio in MHO subjects (30). The adiponectin/leptin ratio was reported to be associated with insulin sensitivity (31), and our study in agreement with previous document demonstrated that this ratio contributed to the metabolic syndrome (28, 32).

In current study, approximately reduced levels of adiponectin to 5.75 (μg/ml) and increased levels of leptin to 8.1 (ng/ml) (calculated from ROC curve) would be required to MetS development in our study participants. These cutoffs usually result in marked manifestation in the metabolic abnormalities associated with obesity and MetS formation. Therefore, the levels of leptin achieved in our MHO subjects (11.2 ng/ml) did not explain “healthy obese phenomenon”. In another word, an elevated plasma leptin level alone does not determine metabolic status in obese subjects. All obese patients in our study, regardless of having MetS, generally had elevated level of leptin, fat mass and trunk fat as compared to normal weight metabolic healthy subjects. However, serum adiponectin levels in MHO as well as normal weight metabolic healthy subjects were higher than MUO patients. Although the leptin level itself powerfully predicts MetS, and metabolic risk profile of obese subjects (33), our results suggest that adiponectin level above 5.75 (μg/ml) is critical for achieving the healthy metabolic status in MHO subjects.

Abdominal obesity was accompanied by reduced adiponectin levels (34). The results of our study with regard to adiponectin in consistent with the previous evidence, adding that simultaneous presence of obesity and MetS, and not only obesity, are required for the reduced adiponectin level. The difference in adiponectin levels observed between groups in the present study could explain the “healthy obese phenomenon”. We found that the magnitude of increase in serum adiponectin correlates with resistance to MetS development even among subjects with obesity. Even though the serum adiponectin level correlated with body-mass index, the relationship between the serum adiponectin level and MetS was independent of obesity, and MHO subjects, as well as normal weight metabolic healthy individual also exhibited adiponectin levels above 5.75 (μg/ml).

At present, there is no compelling evidence to suggest that the lower level of adiponectin contributes to the onset of MetS in healthy obese individuals. However, maintenance of adiponectin at high level may improve metabolic status and prevents the MetS formation in obese patients.

Few tools exist to treat collectively the underlying pathophysiology of obese patients with MetS (35). Therefore, the ability to prediction the onset of the disease may constitute a new, clinically relevant approach to implementing preventive lifestyle interventions or prevention the MetS development in high-risk patients who are overweight or obese. Because the reduction of serum adiponectin level correlates with clinical signs and biochemical components of the MetS, measurement of serum adiponectin could become a noninvasive and accessible method of risk assessment for MetS early-onset in healthy obese patients.

## Conclusion

This study introduced adiponectin and leptin as indicator of MetS and obesity respectively.

## Ethical considerations

Ethical issues (Including plagiarism, informed consent, misconduct, data fabrication and/or falsification, double publication and/or submission, redundancy, etc.) have been completely observed by the authors.

## References

[1] Expert Panel on Detection, Evaluation, and Treatment of High Blood Cholesterol in Adults (2001). Executive summary of the third report of the National Cholesterol Education Program (NCEP) expert panel on Detection, Evaluation, and Treatment of high blood cholesterol in adults (Adult Treatment Panel III). JAMA, 285(19):2486–97. 11368702 10.1001/jama.285.19.2486

[2] Eckel RH Grundy SM Zimmet PZ (2005). The metabolic syndrome. Lancet, 365:1415–1428. 15836891 10.1016/S0140-6736(05)66378-7

[3] Gronner M Bosi P Carvalho A (2011). Prevalence of metabolic syndrome and its association with educational inequalities among Brazilian adults: a population-based study. Braz J Med Biol Res, 44:713–719. 21755260 10.1590/s0100-879x2011007500087

[4] Riediger ND Clara I (2011). Prevalence of metabolic syndrome in the Canadian adult population. CMAJ, 183:E1127–34. 21911558 10.1503/cmaj.110070 PMC3193129

[5] Ford ES Giles WH Dietz WH (2002). Prevalence of the metabolic syndrome among US adults: findings from the third National Health and Nutrition Examination Survey. JAMA, 287:356–359. 11790215 10.1001/jama.287.3.356

[6] Gami AS Witt BJ Howard DE (2007). Metabolic syndrome and risk of incident cardiovascular events and death: a systematic review and meta-analysis of longitudinal studies. J Am Coll Cardiol, 49:403–414. 17258085 10.1016/j.jacc.2006.09.032

[7] Alberti KGM Zimmet P Shaw J (2005). The metabolic syndrome—a new worldwide definition. Lancet, 366:1059–1062. 16182882 10.1016/S0140-6736(05)67402-8

[8] Hanley AJ Karter AJ Festa A (2002). Factor Analysis of Metabolic Syndrome Using Directly Measured Insulin Sensitivity The Insulin Resistance Atherosclerosis Study. Diabetes, 51:2642–7. 12145182 10.2337/diabetes.51.8.2642

[9] Bruun JM Lihn AS Verdich C (2003). Regulation of adiponectin by adipose tissue-derived cytokines: in vivo and in vitro investigations in humans. Am J Physiol Endocrinol Metab, 285:E527–E533. 12736161 10.1152/ajpendo.00110.2003

[10] Xydakis AM Case CC Jones PH (2004). Adiponectin, inflammation, and the expression of the metabolic syndrome in obese individuals: the impact of rapid weight loss through caloric restriction. J Clin Endocrinol Metab, 89:2697–703. 15181044 10.1210/jc.2003-031826

[11] Rasouli N Kern PA (2008). Adipocytokines and the metabolic complications of obesity. J Clin Endocrinol Metab, 93(11 Suppl 1):S64–73. 18987272 10.1210/jc.2008-1613 PMC2585759

[12] Shetty GK Economides PA Horton ES (2004). Circulating adiponectin and resistin levels in relation to metabolic factors, inflammatory markers, and vascular reactivity in diabetic patients and subjects at risk for diabetes. Diabetes Care, 27:2450–7. 15451915 10.2337/diacare.27.10.2450

[13] Kassi E Pervanidou P Kaltsas G Chrousos G (2011). Metabolic syndrome: definitions and controversies. BMC Med, 9:48. 21542944 10.1186/1741-7015-9-48 PMC3115896

[14] Matsuzawa Y Funahashi T Kihara S Shimomura I (2004). Adiponectin and metabolic syndrome. Arterioscler Thromb Vasc Biol, 24:29–33. 14551151 10.1161/01.ATV.0000099786.99623.EF

[15] Mantzoros CS Qu D Frederich RC (1996). Activation of β3 adrenergic receptors suppresses leptin expression and mediates a leptin-independent inhibition of food intake in mice. Diabetes, 45:909–914. 8666142 10.2337/diab.45.7.909

[16] Han SH Quon MJ Kim JA Koh KK (2007). Adiponectin and cardiovascular disease: response to therapeutic interventions. J Am Coll Cardiol, 49:531–538. 17276175 10.1016/j.jacc.2006.08.061

[17] Brooks NL Moore KS Clark RD (2007). Do low levels of circulating adiponectin represent a biomarker or just another risk factor for the metabolic syndrome? Diabetes Obes Metab, 9:246–258. 17391150 10.1111/j.1463-1326.2006.00596.x

[18] Özçelik E Uslu S Kebapçı N (2010). Interrelations of serum leptin levels with adrenocorticotropic hormone, basal cortisol and dehydroepiandrosterone sulphate levels in patients with metabolic syndrome. Diabetes Metab Syndr Clin Res Rev, 4:13–17.

[19] Franks PW Brage S Luan JA (2005). Leptin predicts a worsening of the features of the metabolic syndrome independently of obesity. Obes Res, 13:1476–1484. 16129731 10.1038/oby.2005.178

[20] Zhuo Q Wang Z Fu P (2009). Comparison of adiponectin, leptin and leptin to adiponectin ratio as diagnostic marker for metabolic syndrome in older adults of Chinese major cities. Diabetes Res Clin Pract, 84:27–33. 19181412 10.1016/j.diabres.2008.12.019

[21] Jung C-H Rhee E-J Choi J-H (2010). The relationship of adiponectin/leptin ratio with homeostasis model assessment insulin resistance index and metabolic syndrome in apparently healthy Korean male adults. Korean Diabetes J, 34:237–243. 20835341 10.4093/kdj.2010.34.4.237 PMC2932893

[22] Liao YC Liang KW Lee WJ (2013). Leptin to adiponectin ratio as a useful predictor for cardiac syndrome X. Biomarkers, 18:44–50. 23066861 10.3109/1354750X.2012.730550

[23] Yosaee S Esteghamati A Nasab MN (2016). Diet quality in obese/overweight individuals with/without metabolic syndrome compared to normal weight controls. Med J Islam Repub Iran, 30:376. 27493920 PMC4972048

[24] Yosaee S Khodadost M Esteghamati A (2017). Metabolic Syndrome Patients Have Lower Levels of Adropin When Compared With Healthy Overweight/Obese and Lean Subjects. Am J Mens Health, 11:426–434. 27550773 10.1177/1557988316664074 PMC5675274

[25] Dehghan M Merchant AT (2008). Is bioelectrical impedance accurate for use in large epidemiological studies? Nutr J, 7:26. 18778488 10.1186/1475-2891-7-26 PMC2543039

[26] Aguilar-Salinas CA García EG Robles L (2008). High adiponectin concentrations are associated with the metabolically healthy obese phenotype. J Clin Endocrinol Metab, 93:4075–4079. 18682512 10.1210/jc.2007-2724

[27] Doumatey AP Bentley AR Zhou J (2012). Paradoxical hyperadiponectinemia is associated with the metabolically healthy obese (MHO) phenotype in African Americans. J Endocrinol Metab, 2:51–65. 23293696 10.4021/jem95W PMC3534968

[28] Alfadda AA (2014). Circulating adipokines in healthy versus unhealthy overweight and obese subjects. Int J Endocrinol, 2014:170434. 24550983 10.1155/2014/170434 PMC3914459

[29] Koster A Stenholm S Alley DE (2010). Body fat distribution and inflammation among obese older adults with and without metabolic syndrome. Obesity (Silver Spring), 18:2354–2361. 20395951 10.1038/oby.2010.86 PMC3095947

[30] Labruna G Pasanisi F Nardelli C (2011). High Leptin/Adiponectin Ratio and Serum Triglycerides Are Associated With an “At-Risk” Phenotype in Young Severely Obese Patients. Obesity (Silver Spring), 19:1492–1496. 21183936 10.1038/oby.2010.309

[31] Finucane F Luan J Wareham N (2009). Correlation of the leptin: adiponectin ratio with measures of insulin resistance in non-diabetic individuals. Diabetologia, 52:2345–2349. 19756488 10.1007/s00125-009-1508-3 PMC2759015

[32] Labruna G Pasanisi F Nardelli C (2009). UCP_1_ -3826 AG+GG genotypes, adiponectin, and leptin/adiponectin ratio in severe obesity. J Endocrinol Invest, 32:525–9. 19474520 10.1007/BF03346500

[33] García-Jiménez S Bernal Fernández G Martínez Salazar MF (2015). Serum leptin is associated with metabolic syndrome in obese Mexican subjects. J Clin Lab Anal, 29:5–9. 24659484 10.1002/jcla.21718 PMC6807173

[34] Després JP Golay A Sjöström L (2005). Effects of rimonabant on metabolic risk factors in overweight patients with dyslipidemia. N Engl J Med, 353:2121–2134. 16291982 10.1056/NEJMoa044537

[35] Després J-P (2001). Drug treatment for obesity: we need more studies in men at higher risk of coronary events. BMJ, 322:1379–80. 11397730 10.1136/bmj.322.7299.1379 PMC1120461

